# Point-of-care G6PD diagnostics for *Plasmodium vivax* malaria is a clinical and public health urgency

**DOI:** 10.1186/s12916-015-0531-0

**Published:** 2015-12-14

**Authors:** J. Kevin Baird

**Affiliations:** Eijkman-Oxford Clinical Research Unit, Jalan Diponegoro No. 69, Jakarta, Indonesia; Centre for Tropical Medicine and Global Health, Nuffield Department of Medicine, University of Oxford, Oxford, UK

**Keywords:** *Plasmodium vivax*, Hypnozoite, Relapse, Primaquine therapy, G6PD deficiency, Diagnostics, Acute hemolytic anemia

## Abstract

Malaria caused by *Plasmodium vivax* threatens over 2 billion people globally and sickens tens of millions annually. Recent clinical evidence discredits the long-held notion of this infection as intrinsically benign revealing an often threatening course associated with mortality. Most acute attacks by this species derive from latent forms in the human liver called hypnozoites. Radical cure for *P. vivax* malaria includes therapy aimed both at the acute attack (blood schizontocidal) and against future attacks (hypnozoitocidal). The only hypnozoitocide available is primaquine, a drug causing life-threatening acute hemolytic anemia in patients with the inherited blood disorder glucose-6-phosphate dehydrogenase (G6PD) deficiency. This disorder affects 400 million people worldwide, at an average prevalence of 8 % in malaria-endemic nations. In the absence of certain knowledge regarding the G6PD status of patients infected by *P. vivax*, providers must choose between the risk of harm caused by primaquine and that caused by the parasite by withholding therapy. Resolving this dilemma requires the availability of point-of-care G6PD diagnostics practical for use in the impoverished rural tropics where the vast majority of malaria patients seek care.

## Background

Imagine the clinical importance of a diagnosis that permits safe delivery of a life-saving therapy that is also capable of taking the life of one in ten patients. That diagnosis protects patients at risk of harm by exclusion from treatment and permits the safe treatment of those not excluded. Healthcare providers in the developed world would appropriately view such diagnostic capacity as compulsory, and the clinical consequences of its absence – illness or death caused by the disease or its treatment – as overt malpractice. Over the past six decades, since licensure of primaquine for anti-relapse therapy of malaria caused by *Plasmodium vivax*, safe access to primaquine has been denied by the absence of capacity to diagnose glucose-6-phosphate dehydrogenase (G6PD) deficiency in the impoverished rural tropics.

Therapy of acute *P. vivax* malaria requires two distinct classes of antimalarial drugs: blood schizontocidal and hypnozoitocidal, arresting the acute attack and preventing future recurrent attacks stemming from dormant liver stages called hypnozoites, respectively. The 8-aminoquinoline primaquine is the only therapeutic option for hypnozoitocidal treatment and it invariably provokes acute hemolytic anemia in patients having inherited G6PD deficiency. The early discoverers of G6PD deficiency as the basis of “primaquine sensitivity” in some patients (in 1956) did so with small numbers of otherwise healthy African-American men and they characterized hemolysis as mild and self-limiting [1]. Understanding of the enormous diversity of G6PD deficiency came later, including the common occurrence of variants of relatively extreme sensitivity to primaquine. One reported case of primaquine-induced acute hemolytic anemia in a 13-year-old Indonesian boy having Vanua Lava variant represents extreme primaquine sensitivity: After receiving the fifth of 0.5 mg/kg daily primaquine doses, he reported to clinic cyanotic, dyspnic, and icteric with pronounced hemoglobinuria; his pre-treatment hemoglobin of 12.4 g/dL had fallen to 7.2 g/dL at presentation, and upon admission to an intensive care unit a few hours later, it registered at 5.6 g/dL [2]. Emergency blood transfusions in this instance likely spared the boy’s life. Vanua Lava-like variants of G6PD deficiency (in terms of severity of enzymatic disability) dominate across the Mediterranean, and Southwestern, Southern, and Southeastern Asia, where about 80 % of the global burden of *P. vivax* occurs [3]. Primaquine therapy against relapse in the absence of close clinical monitoring or knowledge of G6PD status should be considered inherently dangerous to any patient. Today the vast majority of patients infected by *P. vivax* have almost no access to clinical monitoring or G6PD screening.

The basis of this problem is neglect, by the medical, scientific and public health communities, the history of which has been described elsewhere [1], as has that of the neglect of *P. vivax* malaria in general [4]. The erroneous assignment of an intrinsically benign and non-life-threatening character of *P. vivax* lies at the core of these multi-factorial and complex problems [5] causing the lack of access to therapy against relapse to have been viewed as inconsequential in endemic areas. In 1981, the malaria treatment guidelines from the World Health Organization (WHO) expressed, for example, “It is doubtful if radical treatment of vivax malaria is necessary if the patient lives in an endemic area where … reinfection is likely” [6].

This withholding of primaquine seemed a reasoned weighing of therapeutic risk and benefit. Standard doses of primaquine against relapse causes acute hemolytic anemia in G6PD-deficient patients, sometimes leading to death [7]. Avoidance of the lethal risk of primaquine for what was perceived as a non-threatening infection dominated strategic thinking by clinical and public health communities. Likewise, the scientific community failed to view safe access to primaquine therapy as a problem in need of solving, either by replacing primaquine or developing G6PD diagnostics suited to where most malaria patients live. This *status quo* of the acceptability of poor access to anti-relapse therapy persisted over sixty years. Very recently, however, these communities have awoken to the need to solve the therapeutic dilemma of primaquine, G6PD deficiency and *P. vivax* malaria. This awareness stems from three key realizations: 1) *P. vivax* is a dangerous infection; 2) its hypnozoites represent a dominant source of acute attacks in endemic areas; and 3) G6PD deficiency renders attacking that reservoir with primaquine an exceedingly dangerous therapeutic endeavor.

## The World Health Organization responds

In 2015, the WHO Global Malaria Program acted decisively to begin removing the barriers to primaquine access for patients infected by *P. vivax*. Both the *Global technical strategy for malaria 2016–2030* [8] and the *Control and elimination of* Plasmodium vivax *malaria: A technical brief* [9] express explicitly the lethal threat of *P. vivax* malaria and the necessity of radical cure with each diagnosis of that infection. Further, recognizing the importance of G6PD diagnostics, by removing the primary barrier of access to that treatment, the WHO convened an Evidence Review Group to examine available technology and devices [10].

The only proven hypnozoitocidal (anti-relapse) drugs are 8-aminoquinolines, all of which provoke hemolytic anemia in G6PD-deficient patients [7]. Thus, identifying appropriate G6PD diagnostic devices and mobilizing them to the periphery of healthcare delivery represents the only possible short-term response to the therapeutic dilemma of *P. vivax* malaria. The clinical and public health threat imposed by the hypnozoite reservoir of that species, now residing in endemic communities almost entirely unmolested by primaquine, underpins the urgency of doing so.

## Hypnozoite reservoir

Relapse behaviors by *P. vivax* vary with geographic distribution in terms of the frequency, timing and multiplicity of secondary attacks [11]. Relapse behaviors of *P. vivax* from the Southwest Pacific represent the most aggressive: almost all infected quickly suffer a first relapse (within 3 weeks of patency of the primary attack) and go on to endure five or more attacks at approximately 2-month intervals, for as late as 4 years following primary infection [12]. These relapse behaviors dominate the strains of Southeast Asia as well, and at least some strains in Africa and the Americas behave similarly. Cohorts of patients diagnosed with *P. vivax* and not treated with primaquine experience an incidence density of first relapse at four to five infections/person-year [13] – approximately the same as *P. falciparum* incidence in much of holoendemic Sub-Saharan Africa. Several studies indicate that hypnozoite-borne attacks dominate over mosquito-borne primary attacks in endemic communities [14, 15]. This dominance largely explains the relatively poor impact of conventional methods of malaria control on endemic *P. vivax* [16]. The broad lack of access to primaquine therapy drives the continuous streaming of repeated clinical attacks and onward transmission from the hypnozoite reservoir.

## Point-of-care G6PD diagnostics

The availability of G6PD diagnostic capacities practical for use in the impoverished rural tropics would protect the minority of residents vulnerable to primaquine therapy. However, the dominating health dividend delivered by such capacity would be realizing the enormous clinical and public health benefits of primaquine therapy for the G6PD-normal majority. Moreover, the great promise of tafenoquine, an efficacious single-dose hypnozoitocidal therapy nearing licensure [17], will also require G6PD screening against the hemolytic toxicity of that 8-aminoquinoline compound.

Experts have described the target product profile (TPP) of point-of-care G6PD diagnostics for screening prior to primaquine therapy [10, 18]. Such a kit must be relatively inexpensive, simple to use and interpret, require no laboratory equipment or skills, be useful at ambient tropical temperatures (>30 °C), and require no cold chain in distribution or storage. Further, the kit must exhibit perfection in negative predictive value (NPV), effectively an estimate of its ability to allow safe administration of primaquine therapy (absence of false normal testing). The current standard for screening G6PD – the fluorescent spot test intended for laboratory use – fails on almost all of those essential criteria.

Only two point-of-care G6PD diagnostic kits are commercially available in 2015: BinaxNOW G6PD™ (Alere, Orlando, FL, USA) and CareStart™ G6PD (Access Bio, Somerset, NJ, USA). BinaxNOW G6PD™ performs satisfactorily [19–21], but it fails the TPP in terms of cost (approximately $15/test) and ambient tropical temperature usage (use above 25 °C strictly prohibited by the manufacturer). CareStart™ G6PD has thus far met essential cost ($1.50/test) and performance characteristics [22–25]. BinaxNOW G6PD™ has been registered and licensed with the US Food Drug Administration, but CareStart™ G6PD has not, and neither product is registered as a prequalified diagnostic device by the WHO. This imposes a significant barrier to kit access by national malaria control programs.

Those two commercial products no doubt represent the leading edge of point-of-care G6PD diagnostics yet to come. Both emerged from private entrepreneurial initiative and capital rather than a supported research agenda bearing development costs and risk. Public investments in research on G6PD diagnostics would no doubt yield improved technologies and devices suited to this specific application guided by a critical TPP. For the present, no other kits exist, however, and awaiting improvements would deny very many millions suffering *P. vivax* malaria safe access to primaquine therapy. That should not be considered an ethically grounded option.

## Conclusions

The availability of devices permitting safe and effective therapy against a life-threatening infection by an otherwise dangerous drug should be heralded as a great stride forward in mitigating the harm done by *P. vivax* or its therapy with primaquine. The ability of national malaria control programs to adopt and integrate G6PD screening capacity into their healthcare delivery systems (to the very periphery) hinges upon the endorsement, encouragement and direct support of international agencies and civil societies. Some authorities, however, consider the point-of-care G6PD diagnostics inadequately vetted or validated and passively await additional data. This posture seems to disregard the current standard of care for G6PD diagnostics in the impoverished rural tropics – the malpractice of no capacity whatsoever.

Understanding this problem requires acknowledging a deeply harmful *status quo* for chemotherapy of *P. vivax* malaria that must be actively and thoroughly discarded. Aggressively rolling out practical point-of-care G6PD diagnostics to the peripheries of healthcare delivery directly enables acting upon the WHO “call-to-arms” against the hypnozoite reservoir. In light of the clinical and public health stakes, the available data on the CareStart™ G6PD kit and its satisfactory fit to the expert TPP should embolden the decisions and actions needed to mobilize it to where needed as matter of urgency. Broad success at doing so promises otherwise inconceivable gains against the global burdens of morbidity and mortality imposed by a pernicious *P. vivax* problem.

## Abbreviations

G6PD: glucose-6-phosphate dehydrogenase
NPV: negative predictive value
TPP: target product profile
WHO: World Health Organization
